# Application of Rapeseed Oil Cake from Biodiesel Production in Methane Co-Digestion with Microalgal Biomass

**DOI:** 10.3390/ma18194542

**Published:** 2025-09-30

**Authors:** Małgorzata Hawrot-Paw, Wiktoria Drzewicka

**Affiliations:** Department of Renewable Energy Engineering, West Pomeranian University of Technology in Szczecin, Pawla VI 1, 71-459 Szczecin, Poland

**Keywords:** microalgal biomass, methane co-fermentation, rapeseed oil cake, biogas, bioenergy

## Abstract

This study aimed to evaluate the potential benefits of co-digesting rapeseed oil cake, a by-product of biodiesel production, with microalgal biomass. Anaerobic fermentation was carried out under mesophilic conditions using various doses of press residue as a co-substrate. The results demonstrate that the addition of rapeseed oil cake enhances biogas production. The highest biogas yield was achieved during co-digestion with 1 g VS·L^−1^ of microalgal biomass and 0.5 g VS·L^−1^ of rapeseed oil cake. The average methane content in the biogas was 62.42%. The average hydrogen sulfide concentration ranged from 400 to 700 ppm. The maximum energy yield of 3.76 kWh·kg^−1^ DM was obtained from co-digesting microalgal and rapeseed oil cake biomass in a 2:1 ratio.

## 1. Introduction

Biomass is a valuable energy resource used in various sectors, such as chemical energy, electricity, and heat [1]. To extract this energy, biomass often needs to undergo various conversion processes. There are many advanced methods of biomass conversion, which are categorized into three main types: thermochemical, physicochemical, and biochemical processes [2,3].

Thermochemical conversion methods use heat and chemical processes. Combustion is the direct burning of biomass in the presence of oxygen to produce heat, which can be used for heating or to generate electricity through steam turbines [4]. Gasification converts biomass into syngas at high temperature with a controlled amount of oxygen or steam [5]. Syngas is a mixture of hydrogen, carbon monoxide, and carbon dioxide, and it can be used for power generation or as a chemical feedstock. Pyrolysis decomposes biomass at high temperatures in the absence of oxygen to produce bio-oil and biochar [6]. Bio-oil can be upgraded to liquid fuels [7], and biochar can be used as a soil amendment [8]. Hydrothermal liquefaction uses subcritical water at high pressure and moderate temperature to produce bio-crude oil, which can be refined into various fuels [9]. Each method has advantages and limitations, the choice of method often depends on the type of biomass, desired end-products, cost, and environmental factors.

Physicochemical methods combine physical and chemical processes to convert biomass into biofuels [10]. Mechanical operations, such as pressing or grinding, are used to extract oils [11]. These oils are converted to biodiesel by transesterification with an alcohol and a catalyst, yielding biofuel and glycerol [12]. Soybean, palm, and sunflower oil are used for biodiesel production, but in Europe, rapeseed is the main feedstock [13]. Pressing produces rapeseed oil cake as a by-product. It contains significant amounts of protein and residual oil [14]. Its chemical composition makes it suitable as a substrate in fermentation during biochemical conversion. These methods use microorganisms to break down biomass structures. Sugars from cellulose and hemicellulose hydrolysis can be fermented to ethanol or other biofuels [15]. Combined with other organic components, they are also a suitable substrate for anaerobic fermentation [16]. All pathways have known limitations. Thermochemical routes require high temperatures, as well as significant gas-cleaning and capital costs, whereas biochemical routes are time-intensive and less effective for recalcitrant biomass [17]. In anaerobic digestion (AD), microorganisms convert organic materials to methane and carbon dioxide [18]. The biogas can be used for heat and power or as a biofuel [19].

Anaerobic digestion is a proven and established technology. The digestion process includes four phases [20]. During hydrolysis, complex organic molecules, such as carbohydrates, proteins, and fats, are broken down by hydrolytic bacteria into monosaccharides, disaccharides, amino acids, and fatty acids. Acidogenesis then converts these volatile fatty acids, alcohols, hydrogen, and carbon dioxide. During acetogenesis, acetogenic bacteria and some archaea convert these intermediates into acetic acid, hydrogen, and carbon dioxide. Methanogenic archaea convert this by-product, in the methanogenesis phase, into methane and carbon dioxide, forming biogas [21,22]. Combining different substrates (co-digestion) can balance nutrients, enhance microbial activity, and increase biogas yield [23]. Diverse substrates can buffer pH changes and other inhibitory conditions [24]. Complementary substrates can also improve process efficiency [25]. Anaerobic digestion is sensitive to inhibitors and operational upsets that depress methane formation. Key constraints include free-ammonia inhibition from nitrogen-rich loads, sulfide from sulfur-containing substrates, and toxicity from some metals or organics [26]. Stable operation depends on loading, pH, and temperature control.

Microalgae can produce a high biomass per unit area due to fast growth. As AD substrates, they offer several advantages. Some microalgae species have high lipid content, which can increase methane production during AD [27]. Microalgae can capture and utilize CO_2_ from industrial emissions, contributing to carbon sequestration efforts [28]. There are also some challenges associated with such biomass. Rigid cell walls can resist microbial degradation, requiring pretreatment to enhance digestibility [29]. Moreover, microalgal biomass often has a low carbon-to-nitrogen (C/N) ratio, which can lead to ammonia inhibition [30]. Co-digestion with carbon-rich substrates can balance C/N ratio and support microbial activity.

Not all substrates are compatible with microalgal biomass. Substrate selection and dosing are required to maximize synergy. Using rapeseed oil cake supports circular economy goals. Rapeseed oil production generates large amounts of press cake. Although it is used as animal feed, utilization is limited by declining livestock numbers [31]. Alternative valorization is therefore needed, including energy use. We hypothesize that the use of rapeseed cake as a co-substrate could increase the efficiency of biogas production compared to single-substrate fermentation.

## 2. Materials and Methods

### 2.1. Materials

**Figure 1:**
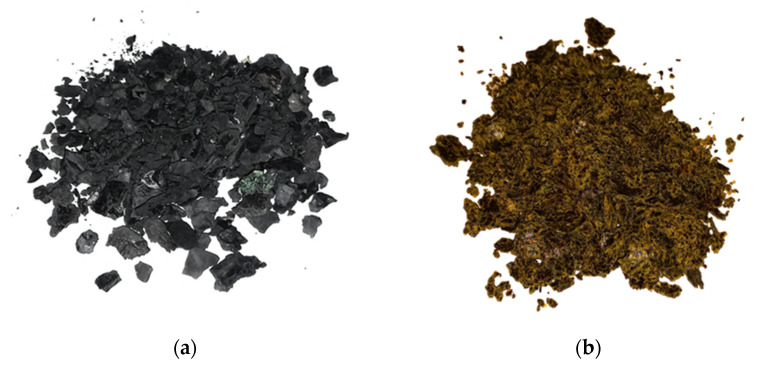
Material used for co-digestion: (**a**) microalgal biomass and (**b**) rapeseed oil cake. Images are illustrative and not to scale.

For this study, we used rapeseed press cake (a biodiesel by-product) and the microalgal biomass of the *Scenedesmus* strain (Figure 1) from our own cultivation system. Rapeseed oil cake was produced by cold pressing with a screw press. Seed moisture before pressing was 5%. *Scenedesmus* was cultivated in vertical 100 L tubular photobioreactors for 10 days on an F/2 medium. LED illumination was 300 µmol m^−2^ s^−1^ with an 18/6 h light/dark cycle. Aeration was 240 L·min^−1^. After cultivation, the microalgal biomass was concentrated by centrifugation, and the microalgal paste was then dried at 35 °C to minimize loss of crucial cellular components.

### 2.2. Experimental Setup

The experiment tested four doses of substrates introduced into the digester:

- 1 g of VS·L^−1^ microalgal biomass—control sample A (C);
- 1 g of VS·L^−1^ microalgal biomass and 0.25 g of VS·L^−1^ rapeseed oil cake (A + W25);
- 1 g of VS·L^−1^ microalgal biomass and 0.50 g of VS·L^−1^ rapeseed oil cake (A + W50);
- 1 g of VS·L^−1^ microalgal biomass and 0.75 g of VS·L^−1^ rapeseed oil cake (A + W75).

A moisture analyzer (AXIS ATS60, Gdansk, Poland) was used to determine the dry mass of the substrate. The materials were dried at 105 °C. Volatile solids were determined by PN-EN 12879:2004 [32]. Substrates were ground with a laboratory mill before being introduced to the digester. The anaerobic digestion process was carried out in a digester with a total volume of 115 L. A paddle mixer provided the substrate distribution inside the digester. The stability of the process parameters was controlled using temperature, pH, and redox potential sensors. Fermentation was carried out under mesophilic conditions at 37 °C. The temperature was stabilized by a water jacket and thermoelectric coolers (TECs).

The biogas composition was measured with a multigas analyzer (MRU OPTIMA BIOGAS, Neckarsulm, Germany): methane content (CH_4_), carbon dioxide (CO_2_), and the calorific value (expressed in MJ·m^−3^). The hydrogen sulfide (H_2_S) concentration was also determined. The amount of biogas was determined using a water meter connected directly to the digester. Quantitative and qualitative analyses of the biogas were performed once daily. We report the biomass energy efficiency as the biochemical in the methane per unit of volatile solids. The energy efficiency (E_e_) was calculated from the following equation [33]:

(1) $$E_{e}\;=\;\frac{Q\cdot C_{v}}{VS},$$

where

E_e_—the energy efficiency of microalgal biomass [kWh·kg^−1^ VS];

Q—the volume of produced biogas [L];

C_v_—the unit calorific value of the produced biogas [kWh·L^−1^];

VS—the mass of converted microalgae [kg VS].

HRT was set to 7 days to match the operating conditions of the full-scale mesophilic digester used for the experiments. This study was designed to evaluate the early conversion and volumetric performance under high-throughput operation. Biogas volume and composition were measured every 24 h on Days 1–4 and Day 7.

## 3. Discussion of Results

### 3.1. Biogas Production

**Figure 2:**
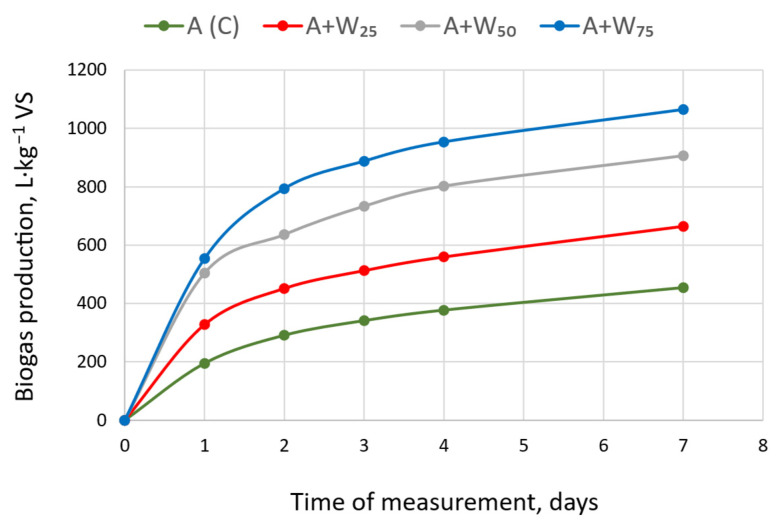
Cumulative biogas yield from anaerobic digestion at different rapeseed oil cake concentrations.

Co-digestion with rapeseed oil cake increased biogas yield (Figure 2). During mono-digestion of the *Scenedesmus* biomass, 454 L·kg^−1^ VS of biogas was obtained compared to a maximum of 505 L·kg^−1^ VS in previous studies for *Arthrospira platensis* [33]. This confirms that the biogas production potential depends on the microalgae strain [29,34], as well as on its cell wall and biochemical structure [27]. Klassen et al. [35] note the importance of cultivation conditions for AD efficiency. Nitrogen-limited biomass can yield more biogas with greater stability than nitrogen-replete biomass. The available data indicate a positive effect of the co-digestion of microalgae with other substrates [36,37], and this is confirmed by the results of the present study. With 25% rapeseed oil cake, the biogas yield increased to 532 L·kg^−1^ VS. At 50% and 75%, cumulative biogas yields were 604 L·kg^−1^ VS and 602 L·kg^−1^ VS, respectively. Pan et al. [38] observed a 2.04–26.86% increase in biogas yield during the co-digestion of food waste with microalgae. Dębowski et al. [39] showed that the co-digestion of *Platymonas subcordiformis* with corn silage and manure resulted in 1.058.8 ± 25.2 L·kg^−1^ VS of biogas with 40% microalgae. This was a significant increase in yield compared to monosubstrate anaerobic digestion. Rapeseed oil cake shows rapid early hydrolysis relative to many agricultural substrates [40]. In our co-digestion trials, adding 25–75% rapeseed oil cake increased biogas production without extending the lag phase, and this is consistent with reports that co-digestion improves degradability and kinetics [41,42]. Rapeseed oil cake supplies readily hydrolysable proteins and residual lipids, which boost early conversion. It contains up to 40% protein depending on the pressing process conditions and 16%-to-20% oil concentration [43,44]. However, excess nitrogen and long-chain fatty acids can inhibit AD if present in excessive amounts [45,46]. Our loading remained below inhibitory ranges for the AD microbial community.

Short HRTs can be appropriate in continuous mesophilic digestion when solids retention and microbial adaptation prevent biomass washout. Stable operation has been reported at about 7 days for waste-activated sludge [47] and at 10 days for microalgae co-digestion in semi-continuous CSTRs [48]. In our study, there was no lag phase, and the biogas volumes exceeded algal mono-digestion within 7 days.

### 3.2. The Content of Methane and Other Gases

**Figure 3:**
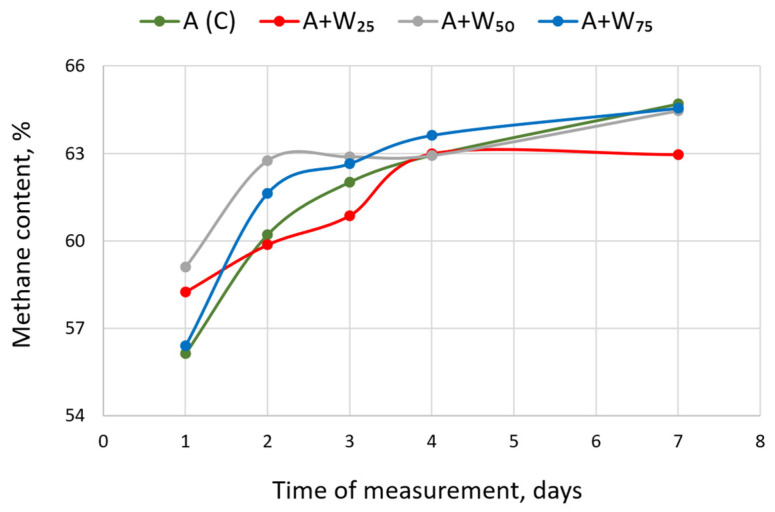
The percentage of methane in biogas at different concentrations of the mixed substrate.

The percentage of the methane content is presented in Figure 3. At the beginning, the CH_4_ ranged from 56.13% to 59.10%. During the first two days, the most significant increase in methane content was observed for the A + W50. The lowest biogas methane content at this date was determined for the A + W25 mixed substrate (62.95%). Studies of Pan et al. [38] have shown that the co-digestion of microalgal biomass with food waste can significantly increase methane production compared to the digestion of microalgal or food waste biomass alone. In the present study, in the control object, the methane content increased gradually and remained stable during the experiment. For the mixed substrate, some fluctuations were observed between the second and fourth days of digestion. Walter et al. [49] investigated biomethane production from microalgal biomass and observed that varying substrate amounts can destabilize AD. Under these conditions, the quantitative composition of microbial groups and their enzyme activities changed.

**Figure 4:**
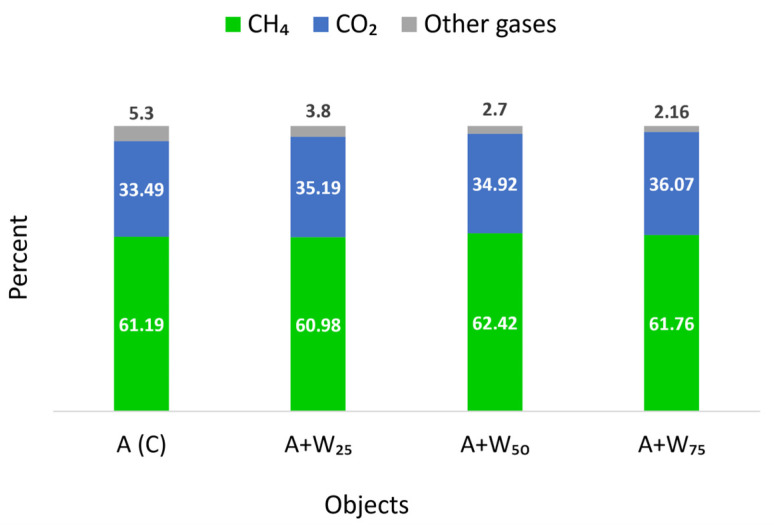
Average biogas composition.

A summary of the average content of components in the biogas is presented in Figure 4. The average content of methane ranged between 60.98% and 62.42%. It was shown that the 50–75% addition of rapeseed oil cake to the microalgal biomass improved methane production. De Castro et al. [50], in a co-digestion study, used a 75:25 (% VS) ratio of food waste to microalgae and obtained a 20–32% increase in methane yield. Ferreira et al. [51] also obtained the highest methane yield for the co-digestion of food waste and microalgae in a ratio of 75:25 (514 mL CH_4_·gVS^−1^). The optimal dose of co-substrates may vary depending on its properties. In the presented study, high rapeseed oil cake content did not provide the expected result, which may suggest some inhibitory effect.

### 3.3. Hydrogen Sulfide Content

**Figure 5:**
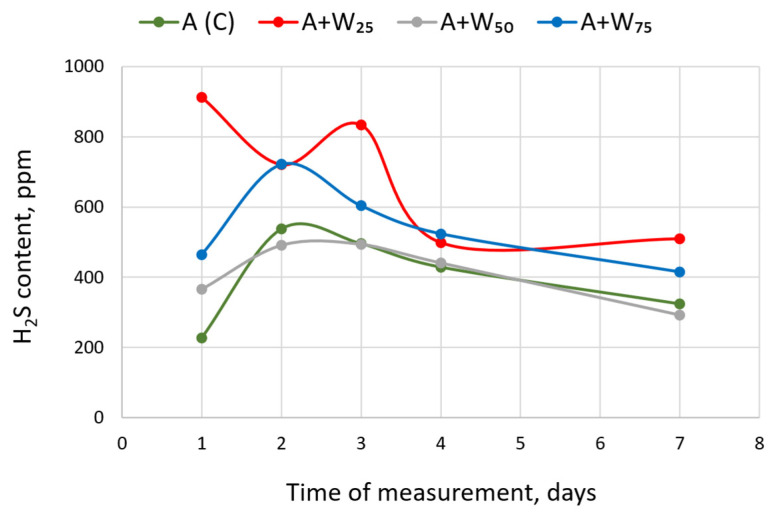
The hydrogen sulfide content in biogas at different concentrations of the mixed substrate.

Hydrogen sulfide is an undesirable biogas component that promotes destructive corrosion [52]. In our analysis, the H_2_S content varied over time (Figure 5). The largest fluctuations were observed for the A + W25 mixed substrate, where values for hydrogen sulfide ranged from 913 ppm on the first day of fermentation to 499 ppm on the last day of the experiment. The lowest H_2_S content in biogas was found in the control and in A + W50. After a Day 2 increase, H_2_S generally decreased thereafter. Similar changes were also observed for the A + W75 load, but the hydrogen sulfide values were higher than in A (C) and in A + W25. The hydrogen sulfide content depends on the degradation of sulfur-containing compounds. Sánchez-Bayo et al. [53] analyzed the elemental composition of the biomass of four species of microalgae and found that the sulfur in this composition accounted for less than 3.5%. H_2_S production during the AD of such biomass would be relatively low. Rapeseed oil cake has a higher sulfur content. According to Jankowski et al. [54], the content of this element ranged from 0.52% to 0.92% of dry biomass, depending on the cultivar. The addition of rapeseed oil cake, therefore, increases the potential sulfur content of the substrate and can raise H_2_S levels in the raw biogas.

**Figure 6:**
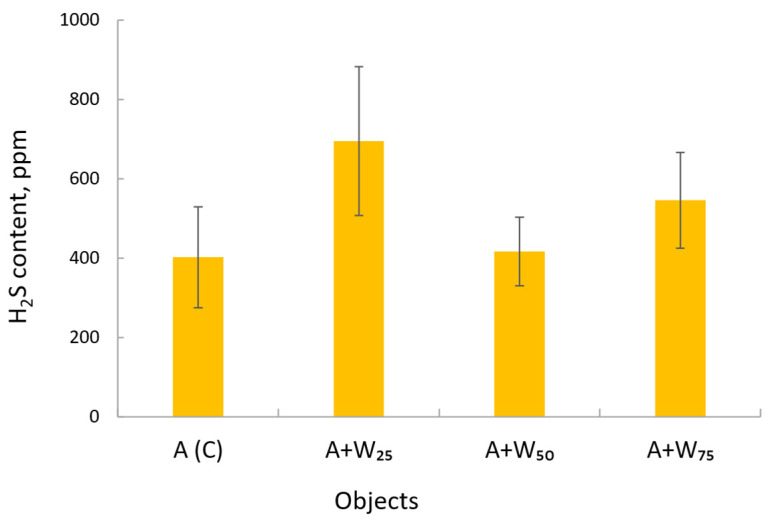
The average hydrogen sulfide content in biogas depends on the substrate type.

The average hydrogen sulfide contents for the loads are shown in Figure 6. The control averaged 402.8 ppm, while A + W25 was the highest at ~700 ppm. For A + W50, the difference from the control values was 15 ppm. With the correct substrate ratio, it was possible to balance carbon and nitrogen [55]. Such conditions support stable microbial activity and reduce H_2_S generation.

Rapeseed oilcake can increase the sulfur through protein-bound sulfur and Brassica-specific glucosinolates with the isothiocyanate products present in rapeseed by-products [56]. Under anaerobic conditions, these components are mineralized to sulfide reduction and can add further sulfide. These routes explain the higher H_2_S with rapeseed oil cake [57,58]. Mitigation options include limiting the press cake fraction (sulfur load), precipitating sulfide as FeS with Fe(II) salts, and applying low rate microaeration to oxidase sulfide to elemental sulfur [58,59].

### 3.4. Methane Fermentation Process Conditions

Methane fermentation was carried out under mesophilic conditions. Table 1 summarizes the operating parameters. The temperature in the digester was set at 37 °C and remained very stable at all times—deviations did not exceed 1 °C. The load’s pH was near neutral and varied at ≤0.1. According to Vongvichiankul et al. [60], the optimal values of the oxidation–reduction potential (ORP) during the methanogenesis phase are around −335.63 ± 28.97 mV. In our study, the measured redox potential ranged from −336 mV to −304 mV, which is consistent with that range and indicates stable operation.

### 3.5. Energy Potential of the Substrate

**Figure 7:**
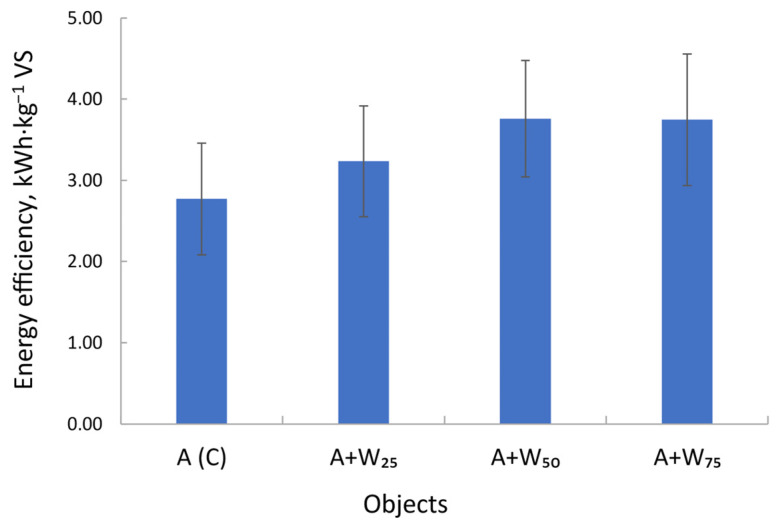
The energy efficiency of biomass conversion under different mixed substrate concentrations.

Figure 7 shows the calculated biomass energy. The results for the A + W50 and A + W75 were highest at 3.76 kWh·kg^−1^ VS and 3.75 kWh·kg^−1^ VS, respectively. The lowest value was observed for the microalgal biomass alone. After seven days, energy recovery was 2.77 kWh per 1 kg VS·L^−1^.

The energy potential of the biomass depends on the microalgal species. For *Arthrospira platensis*, 2.9 kWh per 1 kg has been reported [33]. Cheenakula et al. [61] estimated 1.2–1.5 kWh·kg^−1^ VS for algal–bacterial biomass based on calorific value and organic dry matter.

## 4. Conclusions

Co-digestion allows for the simultaneous treatment of multiple waste streams, improving waste management and lowering disposal costs. Co-digesting microalgal biomass with other organic substrates can increase biogas yield and improve nutrient recovery. In our study, rapeseed oil cake, a by-product of biodiesel production, was an effective co-substrate with microalgae. The highest biogas yield was obtained for mixed substrate A + W50. Rapeseed oil cake did not significantly change the methane percentage; differences were within 1.5%. The hydrogen sulfide level was highest for A + W25, averaging 695 ppm. The lowest hydrogen sulfide concentration was found for the control, at 403 ppm. Rapeseed oil cake can increase H_2_S, although the effect was not linear. The energy efficiency of the biogas from the mixed loads exceeded that from the microalgal biomass alone. The highest energy value, 3.76 kWh·kg^−1^ VS, was obtained using the A + W50 co-substrate mixture. The results suggest that co-digestion of microalgal biomass with rapeseed oil cake at a 2:1 ratio represents the most energy-efficient configuration.

## Figures and Tables

**Table 1:** The parameters monitored during anaerobic digestion.

Variant	Parameter	Day of Measurement				
		1	2	3	4	7
A + C	pH	6.96	6.96	6.96	6.97	6.97
	REDOX [mV]	−307.1	−303.4	−334.4	−334.9	−333.7
	Temperature [°C]	37.18	37.18	37.18	37.18	37.78
A + W25	pH	6.95	6.96	6.96	6.96	6.96
	REDOX [mV]	−321.7	−323.3	−331.3	−336.0	−318.8
	Temperature [°C]	37.78	37.26	37.18	37.18	37.18
A + W50	pH	7.04	7.04	7.04	7.05	7.06
	REDOX [mV]	−316.0	−307.0	−319.0	−314.0	−323.0
	Temperature [°C]	37.00	37.00	37.00	37.00	37.00
A + W75	pH	7.01	7.03	7.04	7.04	7.05
	REDOX [mV]	−305.0	−304.0	−304.0	−308.0	−327.0
	Temperature [°C]	37.00	37.00	37.00	37.00	37.00

## Data Availability

The original contributions presented in this study are included in the article. Further inquiries can be directed to the corresponding author.
